# Functional organization of vestibulospinal inputs responsible for tail postural control in larval *Xenopus*

**DOI:** 10.3389/fneur.2024.1439784

**Published:** 2024-08-16

**Authors:** Gabriel Barrios, Anne Olechowski-Bessaguet, Mathilde Pain, Julien Bacqué-Cazenave, Laura Cardoit, Marie-Jeanne Cabirol, Didier Le Ray, François M. Lambert

**Affiliations:** ^1^Univ Bordeaux, CNRS, INCIA, UMR 5287, Bordeaux, France; ^2^Normandie Univ, Unicaen, CNRS, EthoS, Caen, France; ^3^Univ Rennes, CNRS, EthoS (Éthologie animale et humaine)-UMR 6552, Rennes, France

**Keywords:** motoneuron, larval *Xenopus*, posture, spinal cord, vestibular

## Abstract

In all vertebrates, maintaining trunk posture primarily depends on descending commands originating from brainstem vestibulospinal nuclei. Despite being broadly outlined across species, the detailed anatomical and operational structure of these vestibulospinal networks remains poorly understood. *Xenopus* frogs have previously served as an excellent model for exploring such anatomical and functional aspects in relation to the animal’s behavioral requirements. In this study, we examined the reflex motor reactions induced by vestibular stimulation in pre-metamorphic tadpoles. Our findings indicate that natural vestibular stimulation in the horizontal plane yields greater efficacy compared to stimulation in other planes, a phenomenon replicated in a frequency-dependent manner through specific galvanic stimulation (GVS) of the horizontal semicircular canals. With the exception of a very rostral cluster of neurons that receive vestibular inputs and project to the spinal cord, the overall anatomical segregation of vestibulospinal nuclei in the brainstem mirrors that observed in juvenile frogs. However, our results suggest closer similarities to mammalian organization than previously acknowledged. Moreover, we demonstrated that vestibulospinal cells project not only to spinal motoneurons in rostral segments but also to more distal segments that undergo regression during metamorphosis. Lastly, we illustrated how vestibular-induced spinal reflexes change during larval development, transitioning from tail swim-based activity to rostral trunk bursting responses, likely anticipating postural control in post-metamorphic frogs.

## Introduction

The control of posture performs body stability in space. This integrated motor behavior is a fundamental feature of all motile animals, and constitutes a prerequisite for functional displacements to be achieved (1–3). Postural stabilization results from the multimodal integration of multi-sensory cues together with intrinsic feedforward central commands (4). Amongst sensory modalities, vestibular organs encode head movement and provide the central nervous system with motion-induced inputs that are known to play a major role in regulating spatial body balance in vertebrates (5–7).

In quadrupeds, posture stabilization involves trunk and limb muscles respectively innervated by thoracic and cervical/lumbar motoneurons receiving direct and indirect inputs from vestibulospinal neurons located in brainstem vestibular nuclei (8–13). Although most neural principles of postural adjustment are largely shared among vertebrates (14) it appears that postural control mechanisms are particularly adapted to both biomechanics and environmental constraints specific to each species, as suggested from previous studies in human divers. Besides the traditional “learned process” explanation (15), such functional adaptation could rather be interpreted as postural sensory-motor networks and biomechanical apparatus being intrinsically designed to fulfill their function in a specific environment. However, in most animal species biomechanics are adapted to face environmental constraints. As a consequence, the specific influences of either biomechanics or environment on postural control are usually undistinguishable.

In this context, *Xenopus* constitute very suitable models to study the adequacy of the motor output produced by a sensory-motor neuronal network with the biomechanics, independently of environmental parameters. Because these animals remain exclusively aquatic, the combined modification of neural and effector systems during metamorphosis (16) may provide significant insights onto how neuronal sensory-motor networks are designed specifically related to the animal’s skeletal and muscular arrangement. A recent study in the juvenile *Xenopus laevis* (12) demonstrated that two functionally distinct vestibulo-spinal networks elicited different postural responses respectively based on either trunk muscles activity only or a conjoint action of trunk muscles and hindlimb extensors. Furthermore, these two circuits were activated selectively according to head motion patterns in order to produce the most appropriate reflex response.

*Xenopus* tadpole motility is ensured exclusively by the axial neuromuscular system, which implies a different organization of postural sensory-motor networks compared to frogs. In addition, only the first 10 spinal segments of the larva will be conserved to constitute the adult spinal cord, which suggests a substantially different vestibulospinal organization between segments that will persist and those that will disappear during metamorphosis. Previous works [e.g., (17, 18)] demonstrated that vestibulospinal projections developed at least to rostral spinal segments early during embryonic life (stage 35/36) when tail becomes clearly visible, and that the distribution of brainstem neurons projecting to the spinal cord likely became similar to those of adults as limb buds appear (18, 19). Vestibulospinal functional pathways are well conserved from amphibians to birds and mammals (20) and are organized from two distinct subpopulations so far (18, 21–24). Neurons projecting in the ipsilateral cord originate from the lateral vestibulospinal tract nucleus (LVST) and are mainly located in rhombomeres 4 to 6. Vestibular neurons from the tangential nucleus (TAN) project to the contralateral cord and are restricted to rhombomere 5 and 6. They receive inputs from the entire ipsilateral VIIIth nerve and certainly from contralateral vestibular nucleus through commissural pathways (25). Beyond these simple descriptions, virtually nothing is known about the anatomical organization of vestibulospinal projections and their actions on spinal motor networks in such anguilliform animals. In the present study, to pursue our analysis of the fundamental relationship between sensory-motor organization and behavioral expression, we analyzed the anatomo-functional organization of the vestibulospinal networks regulating postural adjustments in pre-metamorphic *Xenopus laevis*. We performed targeted neuroanatomical studies, as well as electrophysiological recordings of the spinal reflexes evoked using either electrical and natural stimulation of the vestibular sensory system. Our results show that vestibulospinal neurons project to all spinal segments, including those that will not persist in the juvenile. Stimulating these pathways elicits both mono- or polysynaptic inputs onto spinal MNs to generate postural responses adapted to plan-specific head movements, as well as various locomotor-related activities preferentially expressed in mid and caudal tail segments.

## Materials and methods

### Animals

Experiments were conducted on the South African clawed toad *Xenopus laevis* tadpoles obtained from a local aquatic facility in the Institute for Neurodegenerative Disease (University of Bordeaux). Animals were maintained at 20–22°C in filtered water aquaria with a 12:12 h light/dark cycle. Experiments were performed on stage-50 to 55 pre-metamorphic animals, characterized according to external body criteria (26). All procedures were carried out in accordance with, and approved by, the local ethics committee (protocols #2016011518042273 APAFIS #3612).

### Neuronal retrograde and anterograde tracing

Application of neuronal anterograde or retrograde tracer crystals was performed following injection procedures already used in previous publications (12, 23, 24, 27). All wavelength-dependent dextran amine fluorescent dyes [10 kD Alexa dextran amine (AD) 488, 568 and 647 nm; 3 kD tetramethyl-rhodamine dextran amine (RDA); Life Technologies] used in this study are specified in the corresponding figure legend.

Axial motoneurons (MNs) were backfilled from tail myotomes at various distances from the head, differentiating rostral (segments 4–7), medial (segments 12–15) and more caudal (segments 18–22) tail regions (in illustrations, the corresponding myotome numbers are indicated in figure legend). Briefly, consecutive to anesthesia in a 0.05% MS-222 water solution, the skin covering the considered myotomes was dried and a tiny incision was performed allowing the intramuscular application of tracer crystals. Excess dye was washed out with frog physiological saline (120 mM NaCl, 2.5 mM KCl, 30 mM NaHCO_3_, 11 mM glucose, 5 mM CaCl_2_, 1 mM MgCl_2_, pH 7.4). After recovering from anesthesia, larval *Xenopus* were kept in a water tank for at least 48 h to allow tracer migration into MN cell bodies and dendrites.

Vestibulospinal pathways that project onto axial MNs were anatomically deciphered using isolated whole CNS preparations obtained after dissection according to surgery procedures previously described (28, 29). Bilateral vestibulospinal descending neuronal groups were first identified with retrograde labeling. After a tiny unilateral incision, fluorescent dye was applied in a spinal segment on one side, in a ventromedial position that corresponds to axial MNs location in the cord, and tracer migration was allowed for at least 5 h in the dark, in circulating saline at 15–16°C (12, 23) to label brainstem vestibulospinal cell bodies. Concomitant fluorescent labeling of VIIIth cranial nerves allowed the identification of rostro-caudal anatomical markers (vestibular afferents) on cross-sections during confocal imaging. In a series of experiments only the VIIIth nerve anterior ramus was labeled to identify putative connections onto brainstem neurons.

Vestibulospinal terminals onto backfilled axial MNs were revealed with overnight anterograde labeling. Following tiny unilateral incision in brainstem dorsal surface on one side, dye crystals were applied either in rhombomere 4 at the VIIIth nerve level in order to label projections from the lateral vestibulospinal tract nucleus (LVST), or in rhombomere 5–6 at the Xth nerve level to label projections form the tangential nucleus (TAN), respectively.

### Immunofluorescence labeling

After fluorescent tracer migration, isolated CNS preparations were fixed in 4% paraformaldehyde (PFA) at 4°C overnight, then incubated in a 20% [in phosphate-buffered saline (PBS) 0.1%] sucrose solution for 24 h before being embedded in Tissue-Tek (VWR-Chemicals) and frozen at −45°C in isopentane. Fluorescence immunohistochemistry on brainstem and spinal cord 20 μm cross-sections obtained with a cryostat (CM 3050, Leica) was performed afterwards [for complete details, see (30)]. Mouse primary antibodies anti-synapsin (1200, Synaptic Systems) were used to label presynaptic terminals and subsequently revealed with donkey secondary anti-mouse IgG coupled to Alexa Fluor 488 (1200, Thermo Fisher). Samples were incubated with the primary antibody for 24 h at 4°C. After rinsing with PBS, samples were incubated for 90 min at room temperature with the fluorescent dye-coupled secondary antibody. After fast washing in distilled water, cross-sections were mounted in a homemade medium containing 74.9% glycerol, 25% Coon’s solution (0.1 M NaCl and 0.01 M diethyl-barbiturate sodium in PBS), and 0.1% paraphenylenediamide.

### CLARITY-based treatment of larval brainstem tissue

After retrograde vestibulospinal labeling, brainstems were treated for a hydrogel clearing method derived from CLARITY, as extensively described in Cabirol et al. (31). First, brainstems were fixed at 4°C in PBS 0.1 M containing 4% PFA, 4% acrylamide (Merck) and 0.25% VA-044 thermal initiator (Fujifilm, Wako, Germany) for 24–36 h. To perform the hydrogel polymerization, samples were then incubated in a 4% acrylamide/0.25% VA-044 solution (in PBS 0.1 M) for 6 h at 37°C under constant shaking. The brainstem-containing polymerization solution was covered by peanut oil in a tube sealed with parafilm to prevent oxygen entrance. Thereafter, samples were rinsed 3–4 times in PBS-azide (0.1 M PBS and 0.01% sodium azide) at room temperature. After rinsing, samples were incubated at 37°C in a detergent clearing solution (4% sodium dodecyl sulfate in 200 mM boric acid at pH 8.5 with 0.4% lithium hydroxyde monohydrate) for 2 weeks. The solution was replaced every 4 days. For the last step, samples were washed in a PBS-azide solution (PBS 0.1 M with 0.01%sodium azide) at room temperature during 48 h, replaced twice a day. For confocal imaging, brainstems were mounted in a thick chamber, in a PBS-azide solution containing 66% fructose and 20% DMSO for refractive index matching.

### Image acquisition and processing

Cross-sections labeled with fluorescent materials were imaged using a Zeiss LSM900 confocal microscope equipped with 488, 543 and 633 nm laser lines. Multi-image confocal stacks with 1 μm z-step intervals were generated using a 20×/0.75 oil objective and with 0.3 μm z-step intervals using a 60×/1.4 oil objective. Cell population images were obtained by orthogonal projection from multi-image stacks while synaptic apposition images were recorded from single confocal planes. Final images were processed with artificial fluorescent colors using Fiji^1^ and Photoshop (Adobe Systems Inc.) softwares.

### *In vitro* semi-intact and isolated preparations

Animal dissection for *in vitro* preparations and nerve recording procedures were conducted as described extensively in Rauscent et al. (29). Under anesthesia and after viscera and forebrain removal, the brainstem-spinal cord together with the ventral roots (Vr) were dissected out from stage 53–55 tadpoles. *In vitro* experiments were performed in carbogen-bubbled saline, either normal frog saline (see above) or a solution enriched in divalent cations (101.15 mM NaCl, 2.05 mM KCl, 20 mM NaHCO_3_, 11 mM glucose, 11.15 mM CaCl_2_, 3.05 mM MgCl_2_, pH 7.4) in order to raise neuronal spiking threshold and preserve only monosynaptic connections (32). In semi-intact preparations, the two otic capsules were kept intact and attached to the brainstem with the VIIIth nerve. Vr extracellular activity was recorded at a rostral, a medial, and a caudal level (typically at spinal segments 5, 12 and 19) using borosilicate glass suction electrodes (tip diameter, 100 nm; Clark GC 120F; Harvard Apparatus) filled with saline. In some experiments, specific extraocular motor nerves were dissected out and recorded from similarly as spinal roots. Electrophysiological signals were directed through extracellular amplifiers (AM-Systems) and an analog/digital conversion interface (CED 1401; Cambridge Electronic Design) to a computer where signals were stored using the software Spike2 (CED).

### Stimulations of vestibulospinal pathways

In vestibular nuclei stimulation experiments, a ventral hemi-section was performed at C1 level to prevent composite (LVST+TAN) vestibulospinal commands to activate downstream motoneurons. Electrical stimulation of vestibular nuclei consisted of single pulses delivered with a Grass stimulator S88 through a 2 MΩ monopolar stainless electrode (Micro Probe, Inc.). Stimuli (10–15 μs; 5–10 V) were always set with the minimal voltage amplitude capable of eliciting stable motor responses in normal saline. Galvanic vestibular stimulation (GVS) (33) was applied through two 0.76 mm Teflon-coated silver electrodes (AG 25-T; Science Products), the chlorinated tips of which were placed under visual guidance in close proximity to the visible cupula of the horizontal semicircular canal on the two sides of the body. Stimuli were delivered as sinusoidal currents using a linear stimulus isolator (A395; World Precision Instruments) triggered by the Spike2 wave editor. Sinusoidal galvanic currents were applied to both electrodes in phase opposition at frequencies of 0.1, 0.2, 0.5, or 1 Hz, with maximal intensities of ±0.1, 0.25, 0.5, or 1 mA. Thus, sinusoidal GVS consisted of repeated cycles of positive and negative currents, respectively evoking sensory cell excitation and inhibition, oppositely delivered simultaneously on left and right-side horizontal canal cupulas. Natural stimulation of vestibular endorgans was performed using a computer-controlled, motorized stimulation apparatus (Turn-table and Sled; Technoshop COH@BIT, IUT de Bordeaux, University of Bordeaux). The animal was centered in the horizontal rotation and translation axes in order to provide natural activation of horizontal semicircular canals and utricles [see also (12)]. Ten-cycle sequences of sinusoidal stimulation were performed at 1.0 Hz, with amplitudes of ±5–60°/s for rotation. In this apparatus, Vr discharges were recorded with adjusted suction electrodes using EXT 10-2F amplifiers (NPI Electronics). An additional subset of experiments was performed on a computer-controlled hexapod [Physics, Germany (34)]; in Prof. H. Straka lab (Div. of Neurobiology, LMU, Munich) to record Vr activity in response to 10-cycle (0.1 Hz; ±5–10°/s) sequences of sinusoidal roll and pitch head movements.

### Calcium imaging

In semi-intact preparations (*n* = 5 preparations), Calcium Green-1 Dextran Amine crystals (3 kD CGDA; Invitrogen) were injected unilaterally in a rostral spinal segment (same procedure as described above for retrograde tracing) in order to backfill vestibulospinal neurons. After the CGDA migration, the spinal cord was completely transected at the obex to prevent any parasite effect from the spinal activity on vestibulospinal neuron response to the GVS. Calcium imaging experiments were performed in the same way as previously described in Olechowski‐Bessaguet et al. (12). Calcium transients evoked in vestibulospinal neurons by GVS stimulation were optically recorded with an epifluorescence microscope (Olympus BX51WI) and a high CCD camera (QImaging OptiMOS) at a 10-fps image rate (time exposure = 95 ms; image interval = 100 ms) under a x40 water immersion objective (Olympus LUMPlanFLN). Single cell calcium transients were measured within ROI drawn over individual neuron somata (*n* = 20) using MetaFluor (Meta Imaging Series 7.8; Molecular Devices) and expressed as fluorescence changes relative to the fluorescence baseline (∆F/F).

### Data analysis and statistics

Electrophysiological extracellular recordings of Vr discharges were analyzed offline with Spike2 (CED). To analyze the modulation of nerve activity in response to natural vestibular stimulation, ventral root activities were integrated and averaged over 10 successive stimulation cycles. Statistical analysis of electrophysiological data was performed using Prism5 (GraphPad) and OriginPro8 (OriginLab Corporation), and data are given in the text as means and standard errors of the mean (±SEM), unless stated otherwise. In order to respect 3Rs, small animal samples were used, and only non-parametric tests were performed to analyze such linear data. Circular phase analysis was performed using Oriana (Kovach Computing Services). The non-homogeneity of phase distributions was first tested with the Rayleigh test, and subsequent circular statistical analysis was performed only on non-homogenous distribution; no distributions in this study were found homogenous. Similarity between two circular distributions was then investigated using the Watson *U*^2^ test, for which the null hypothesis is that the two distributions are identical, and a significant *p*-value indicates rejection of the null hypothesis. Spectral analysis, which global procedure was detailed in Bacqué-Cazenave et al. (35), was performed to extract principal frequencies from Vr extracellular activities. Briefly, a Fourier transformation as first step decomposed the recorded signal into multiple sinusoidal signals of different frequencies, thereby identifying the principal oscillation frequencies, and a wavelet analysis confirmed the principal oscillation frequencies and reconstructed the corresponding sinewaves. Results were presented as spectrograms showing wavelet power spectrums of each continuous oscillation over recordings’ frequency range, and periodograms illustrating the average power of each oscillation frequency.

## Results

### Synergistic vestibular-induced ocular and spinal reflex responses in tadpoles

**Figure 1:**
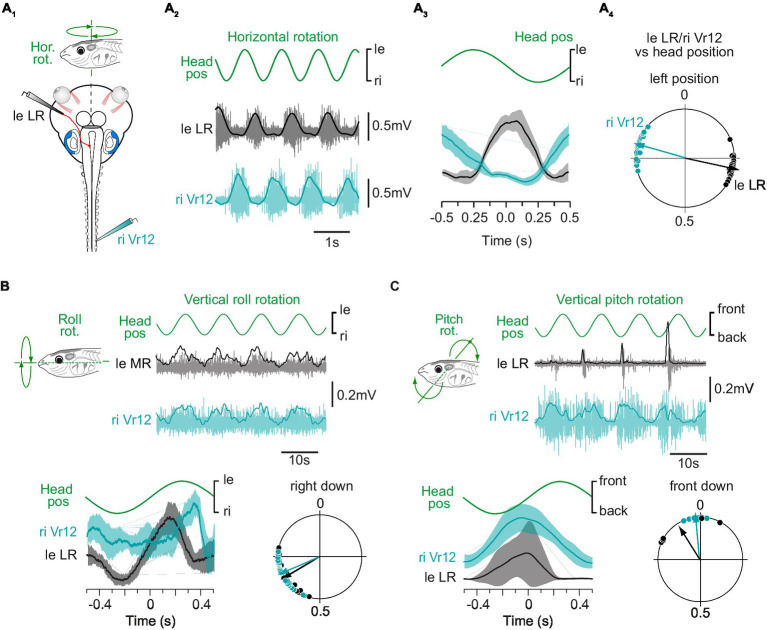
Spinal motor responses to vestibular endorgan natural stimulation. **(A)** Spinal ventral root (Vr; turquoise) and contralateral lateral rectus (LR; grey) nerve activity recorded in a semi-intact preparation **(A**_**1**_**)** in response to sinusoidal head rotations in the horizontal plan **(A**_**2**_**)** and corresponding mean discharges rates **(A**_**3**_**)** and phase relationships **(A**_**4**_**)** relative to head position. Plot in **A**_**3**_ shows mean responses averaged on 10 cycles for one animal. Circular diagram in **A**_**4**_ displays the phase distribution (dots) and mean vector (arrow) for each nerve activity in one typical animal. **(B)** Spinal ventral root and contralateral medial rectus (MR; grey) nerve activity recorded in response to sinusoidal head rolls in the vertical plan and corresponding mean discharge rates (bottom left) and phase relationships (bottom right) relative to head position. **(C)** Spinal ventral root and contralateral LR nerve activity recorded in response to sinusoidal head pitches in the vertical plan and corresponding mean discharge rates (bottom left) and phase relationships (bottom right) relative to head position. In **A–C**, the right ventral root 12 (riVr12) reflex discharge was compared to the well-known vestibulo-ocular response recorded simultaneously in either the left eye lateral or medial rectus motor nerve.

**Figure 2:**
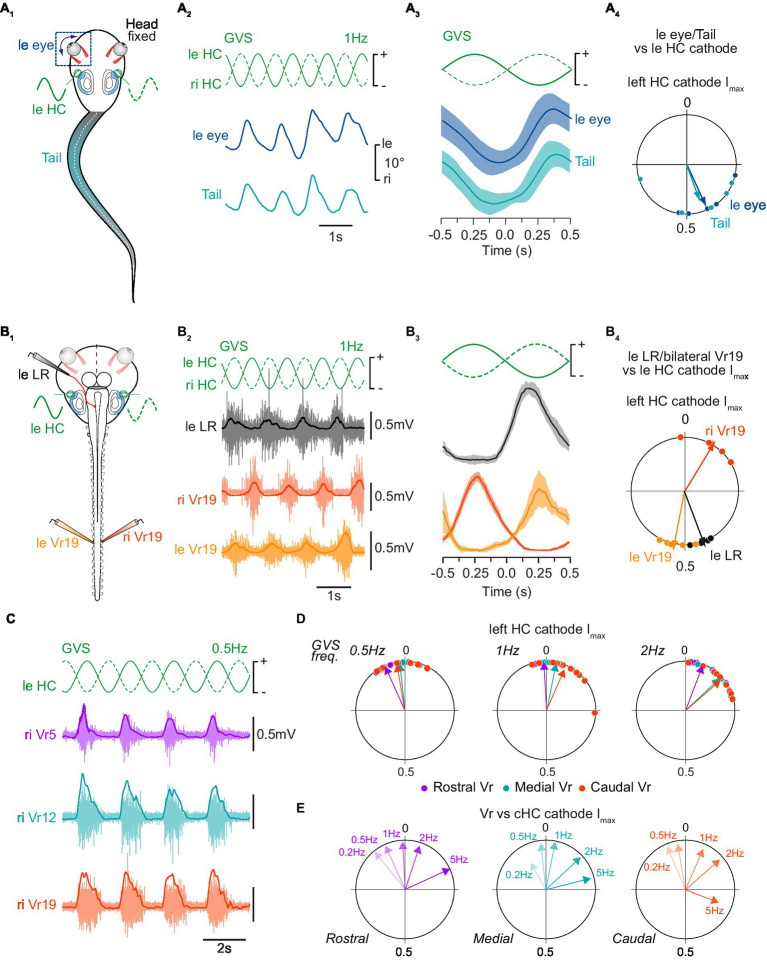
Tail postural and spinal motor responses to galvanic vestibular stimulation. **(A)** Tail postural adjustments (turquoise) and movements of the left eye (blue) recorded in head fixed *in vivo* preparations **(A**_**1**_**)** in response to sinewave galvanic stimulation (GVS) of horizontal canal cupula on left (leHC) and right (riHC) sides **(A**_**2**_**)**, corresponding mean movements relative to the stimulus cycle **(A**_**3**_**)**, and corresponding phase relationships relative to the maximal cathode stimulation current (*I*_max_) of the left horizontal canal cupula (leHC, **A**_**4**_). Plot in **A**_**3**_ shows mean responses averaged on 10 cycles. Circular diagram in **A**_**4**_ displays the mean distribution of phases (dots) and mean vector (arrows) for tail and eye movements. **(B)** Bilateral distal ventral root (leVr19, riVr19; orange) and left lateral rectus (leLR; grey) nerve activity recorded in a semi-intact preparation **(B**_**1**_**)** in response to GVS stimulation of horizontal canal cupula **(B**_**2**_**)**, corresponding mean discharge rates relative to GVS phase **(B**_**3**_**)**, and corresponding phase relationships relative to the maximal left HC cathode current **(B**_**4**_**)**. **(C)** Illustration of concomitant postural motor activity in right rostral (Vr5, purple), medial (Vr12, turquoise) and caudal (Vr19, orange) ventral roots in response to 0.5 Hz GVS. **(D)** Reflex response phase distribution and mean vector for all ventral roots relative to the maximal left cathode current at 0.5 Hz (left circular diagram), 1 Hz (middle circular diagram) and 2 Hz (right circular diagram). **(E)** Mean vectors for each ventral root reflex activity relative to 0.2, 0.5, 1, 2 and 5 Hz GVS sinusoidal *I*_max_ of the contralateral horizontal canal cupula (cHC).

In most species, the vestibulo-ocular reflex (VOR) was better studied than vestibulospinal responses, mainly because VOR relies on the activation of only three pairs of extraocular muscles (5, 6). In larval *Xenopus* as well, both angular and linear VOR were particularly well defined (33, 36–38). Consequently, in the present work both vestibulo-ocular and vestibulo-spinal motor activities were compared in a first set of experiments (Figures 1, 2) to better determine the temporal organization of vestibulospinal reflexes and the synergistic action of vestibular-induced ocular and spinal motor responses.

In pre-metamorphic tadpoles, swimming consists of tail undulations due to alternate axial muscle contractions (27), which causes left-right oscillations of the head in the horizontal plane (39, 40), activating preferentially the horizontal semicircular canals. Such head displacements were reproduced experimentally on a turn-table while extraocular and motor responses from the medial spinal cord were recorded (*n* = 3; Figure 1A). As previously reported, lateral rectus (LR) motor nerve was activated during contraversive head rotations and silent during ipsiversive head turn [left LR activated during right head turn in Figure 1A; see also (38, 41)]. Comparatively, mid-caudal ventral root (Vr12) showed the same discharge pattern as LR nerve during horizontal head rotations (right Vr12 activated during leftward head turn in Figures 1A_2_,A_3_), as succinctly described in a previous study (23). The circular distribution of phases (Figure 1A_4_) for both LR and Vr motor nerve activity relative to the mean stimulus cycle (as illustrated in Figure 1A_3_) confirmed that vestibulospinal reflex responses were produced in phase opposition with the direction of head rotation, likewise VOR responses.

Natural stimulation was also used to investigate the potency of vertical semicircular canal and utricular afferents to trigger reliable spinal reflexes. Low dynamic roll and pitch head rotations are known to activate specifically utricles (38). Conjoint LR and mid-caudal Vr motor nerve activity was recorded during sinusoidal roll (*n* = 8; Figure 1B) and pitch (*n* = 6; Figure 1C) head movements. Both vertical stimulations were able to evoke burst discharges in extraocular and spinal motor nerves, mainly during contraversive head displacements. However, spinal Vr bursts were less temporally defined in response to vertical head rotations than they were in response to horizontal rotation. Therefore, the rest of the study focused on horizontal semicircular canal-evoked spinal reflexes.

GVS stimulation applied on horizontal canal (HC) cells was shown to mimic horizontal rotations of the head (33). In a first set of experiments (*n* = 5; Figure 2A) GVS electrodes were placed bilaterally on head-fixed semi-intact preparations where tail and eyes were preserved intact (Figure 2A_1_). Kinematic analysis of actual eye and tail movements produced in response to GVS demonstrated cyclic ocular and conjoint maximum tail curvature (42) to the opposite side of the HC cupula activated by the cathode current (marked by a “+” in figures; example in Figures 2A_2_,A_3_ show a left eye and tail displacement to the right when the left HC cupula is activated). The circular distribution of phases for eye and tail maximal deflection relative to the mean stimulus cycle (Figure 2A_4_) confirmed that both vestibulo-ocular and vestibulospinal reflex responses occurred simultaneously in the same direction (*p* > 0.5, Watson *U*^2^ test: *U*^2^ = 0.05), with movement onset occurring close to the maximal excitation of contralateral HC sensory cells (eye: 155 ± 31°; tail: 160 ± 52°).

The same sinusoidal GVS was then applied to HC cupulas of *in vitro* isolated brainstem/spinal cord preparations with the otic capsules attached (*n* = 5; Figure 2B_1_), avoiding any putative water displacement artifact that could affect vestibular sensory perception during actual tail movement. In such conditions, left LR motor nerve was activated during excitation of the right HC sensory cells (Figures 2B_2_,B_3_), which thus mimicked rightward head rotation (Figure 1A). Spinal ventral roots also responded to GVS similarly to natural stimulation, with each Vr being activated during excitation of the contralateral HC cells (Figures 2B_2_,B_3_). The circular distribution of phases (Figure 2B_4_) relative to the mean stimulus cycle (as illustrated in Figure 2B_3_) confirmed that reflex bursts in LR nerves (left LR nerve in example illustrated in Figure 2B_4_) peaked close to maximal HC excitation i.e., with a mean phase lag of 70 ± 4° relative to the onset of the contralateral HC excitation. Similarly, Vr reflex bursts were maximal with mean phase lags of 10 ± 7° for left Vr and 27 ± 10° for right Vr, respectively relative to the maximal excitation of contralateral HC cells. As a consequence, reflex activity on Vr19 on the two sides occurred in strict antiphase (Figure 2B_4_; *p* < 0.05, Watson *U*^2^ test: *U*^2^ = 0.225).

GVS at various cycle frequencies (0.2–5 Hz) evoked mostly reliable Vr reflex responses in the rostral, medial and caudal spinal cord, although 0.5, 1 and 2 Hz were the more efficient stimulation frequency to trigger VR responses (an example at 0.5 Hz is illustrated in Figure 2C). Maximal Vr activity occurred at peak excitation of contralateral HC cupula, thus corresponding to a compensatory reflex response aiming at restoring body balance. Whatever the GVS frequency, spinal reflexes were initially evoked in rostral segments in first place and, then, occurred almost synchronously in medial and caudal segments (Figure 2D), although this temporal distribution was only significant at 1 and 2 Hz (paired *F*-test between rostral and either medial or caudal segments: *p* < 0.05; paired *F*-test between medial and caudal segments: *p* > 0.05; *n* = 8). The timing of reflex responses within the stimulus cycle also depended on GVS frequency since response occurrence gradually shifted clockwise while GVS frequency was increased (Figure 2E). However, no statistical differences could be observed between low GVS frequency-evoked mean responses (*p* > 0.05 for *F*-tests pairwise comparing 0.2 and 0.5 Hz GVS frequencies). In contrast, at 1 Hz and above, phases of mean GVS-evoked responses significantly differed from each other and from low frequency-evoked reflexes at both rostral and caudal spinal levels (*p* < 0.05 for all paired *F*-tests); however, in medial segments, the distinction became statistically different only from a 2 Hz GVS frequency.

### Vestibulospinal pathways underlying bodily postural reflexes

Anuran amphibians undergo a full metamorphosis (16) characterized, among other things, by the complete regression of the tail, the posturo-locomotor appendage at embryo and tadpole stages. Only the anterior part of the body is preserved after metamorphosis, and the posturo-locomotor biomechanical system is fully remodeled, with fully adapted vestibulospinal projections organized within the remaining 10 spinal segments (12). To better understand how this adult vestibulospinal arrangement emerged from tadpole organization we first asked whether vestibulospinal inputs were already mainly, if not totally, restricted to the more rostral segments of the tadpole cord.

**Figure 3:**
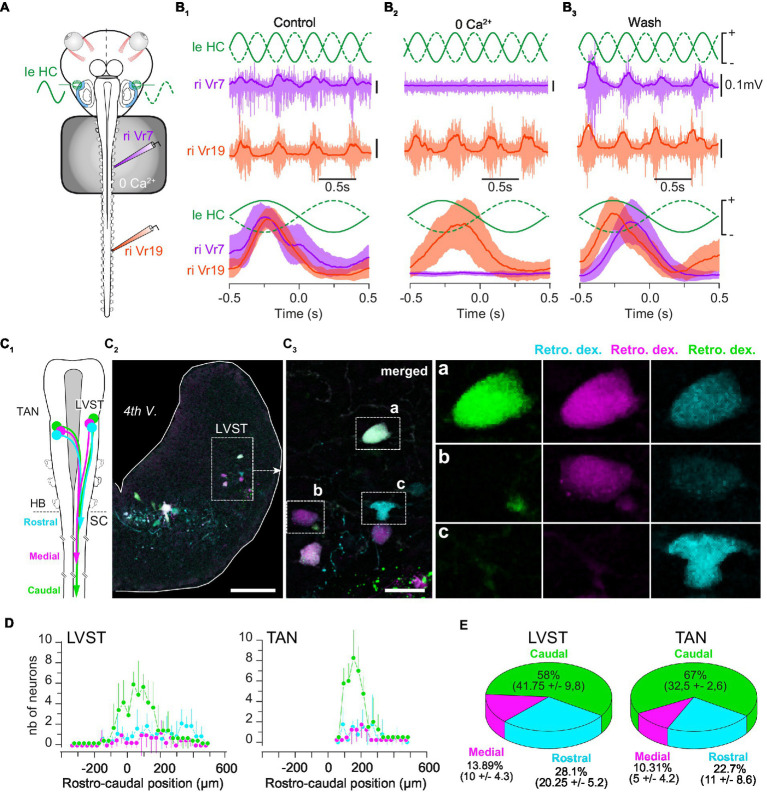
Vestibulospinal neurons project all along the spinal cord. **(A)** Schematic of the semi-intact preparation used to record GVS-evoked rostral and caudal spinal motor activity during bath application of a calcium free (0 Ca^2+^) ringer solution on the rostral cord (segments 1 to 10). **(B)** Example of 1 Hz GVS-evoked reflex motor activity (top) in rostral (Vr5, purple) and caudal (Vr19, orange) spinal ventral roots, and corresponding phase-related discharges averaged over 10 cycles (bottom) before **(B**_**1**_**)**, during **(B**_**2**_**)** and after **(B**_**3**_**)** calcium-free perfusion. **(C)** Sequential retrograde dye application in caudal, medial and rostral spinal hemi-segments **(C**_**1**_**)** revealed LVST neurons in the brainstem **(C**_**2**_**)** projecting directly to caudal spinal segments (triple labeled neurons, inset “**a**” in **C**_**3**_), directly to medial segments (double labeled neurons, inset “**b**” in **C**_**3**_) and to rostral segments (single labeled neurons, inset “**c**” in **C**_**3**_). Similar observation was made for TAN neurons (not illustrated). **(D)** Rostro-caudal distribution (mean ± SD) of LVST (left graph) and TAN neurons (right graph) projecting to caudal (green), medial (pink) and rostral (cyan) spinal segments. The 0 μm position corresponds to the VIIIth nerve rostral entrance in the brainstem. **(E)** Proportion (mean ± SD) of LVST (left pie chart) and TAN neurons (right pie chart) projecting to caudal (green), medial (pink) and rostral (cyan) spinal segments. 4th V., 4th ventricle; LVST, lateral vestibulospinal tract nucleus; TAN, tangential nucleus; Retro. Dex., retrograde dextran-coupled dye; HB, hindbrain; SC, spinal cord; nb, number. Scale bar is 100 μm in **C**_**2**_ and 25 μm in **C**_**3**_.

Sinusoidal GVS was applied *in vitro* to stimulate HC sensory cells while reflex activity was recorded simultaneously from a rostral and a caudal ventral root in normal conditions or when rostral segments only were perfused with a saline deprived of calcium (*n* = 3; Figures 3A,B). As reported above, GVS evoked spinal reflexes at both rostral and caudal levels in control conditions (Figure 3B_1_). In contrast, GVS-evoked rostral responses were reversibly abolished in calcium-deprived saline, without affecting reflex generation in otherwise normally perfused caudal segments (Figures 3B_2_,B_3_). These results functionally demonstrated that in tadpoles, some vestibulospinal neurons projected farther in the spinal cord than the first 10 segments that would be preserved after metamorphosis, suggesting the vestibulospinal organization in tadpole to differ from the one previously described in juveniles (12).

Retrograde fluorescent dyes were used to characterize vestibulospinal projections in the tadpole spinal cord form both LVST and TAN nuclei (*n* = 5; Figures 3C–E). Sequential injection (Figure 3C_1_) of three distinct dyes in segment 19 (caudal; Figure 3C_3_,a), segment 11 (medial; Figure 3C_3_,b) and segment 4 (rostral; Figure 3C_3_,c), respectively, was used to determine the proportion of LVST and TAN neurons projecting in the three different parts of the spinal cord (Figures 3D,E). Analyzing the rostro-caudal distribution of the labeled vestibulospinal neurons in the brainstem failed to demonstrate any somatotopy in either vestibulospinal nucleus, neurons projecting in the rostral spinal cord being intermingled with those projecting in medial and caudal segments (Figure 3D), partly due to our semi-quantitative dextran dye injection method that provided only estimated counts. However, it appeared that a large majority of the labeled neurons in the two nuclei projected in the caudal cord (LVST: 58%; TAN: 67%; Figure 3E), whereas only a small proportion of neurons likely stopped in the medial segments (LVST: ~14%; TAN: ~10%; Figure 3E) or in the more rostral segments (LVST: ~28%; TAN: ~23%; Figure 3E).

**Figure 4:**
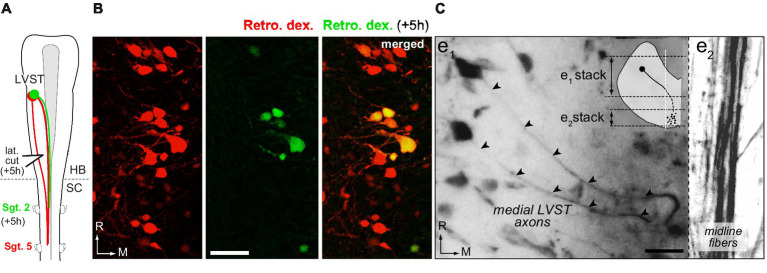
Lateral and medial trajectories of LVST axons in the brainstem. **(A,B)** Sequential application of retrograde dyes in spinal hemi-segments (Sgt.) 5 and 2 with an in-between lateral cut (lat. cut) in the caudal brainstem **(A)** revealed a subpopulation of LVST neurons projecting medially within the brainstem (**B**, double labeled cell bodies). **(C)** Trajectory of LVST axons (arrowheads) going directly to the midline visualized in whole brainstem preparation treated with CLARITY. Two confocal stack projections (see schematic in inset) at two different depths in the same brainstem area respectively illustrate the initial axon courses toward the midline (e1) and the bundle of LVST axons running parallel to the midline. Retro. dex., retrograde dextran dye; HB, hindbrain; SC, spinal cord; R, rostral; M, medial. Scale bar is 20 μm in **B,C**.

Sequential retrograde labeling of LVST neurons was performed in order to identify the LVST neurons which traveled laterally in the brainstem from those directly projecting medially (*n* = 2; Figure 4A). A first fluorescent dye was injected in the 5th spinal hemi-segment (red in Figure 4A), followed 5 h later by the injection of a second dye in the 2nd homolateral hemi-segment (green in Figure 4A). To be able to separate between the two LVST populations, a lateral cut was made in the caudal brainstem on the same side in the time interval between the two dye injections (Figure 4A). Such a procedure revealed a small proportion of LVST neurons (23 ± 8%; Figure 4B) sending their axon towards the brainstem midline, although these axons all remained ipsilateral to their cell body (Figure 4C).

### Spinal motoneurons receive direct vestibulospinal inputs from LVST and TAN nuclei

**Figure 5:**
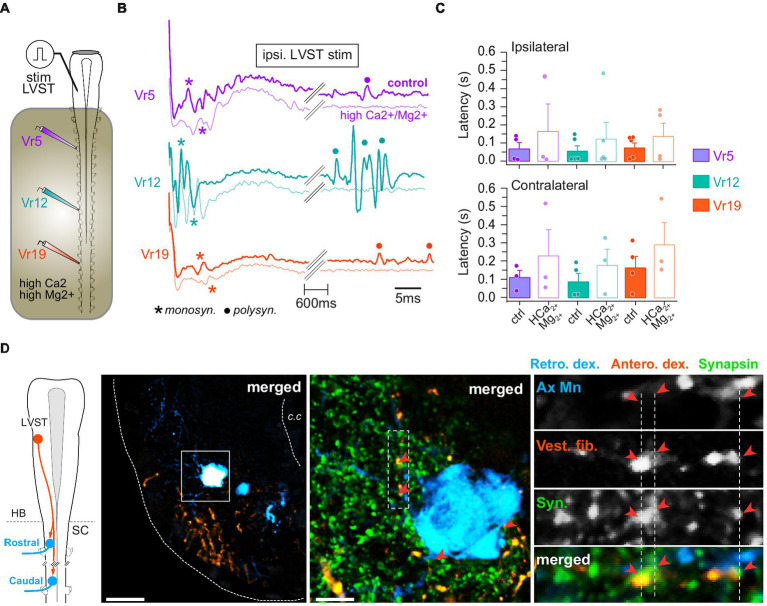
LVST inputs on spinal motoneurons. **(A)** Schematic of the semi-intact preparation used to record spinal ventral root responses to electrical stimulation of the LVST nucleus. **(B)** Trace examples of LVST-evoked motor response in rostral (Vr5, purple), medial (Vr12, turquoise) and caudal (Vr19, orange) ventral roots before and during bath application of Ca^2+^/Mg^2+^-enriched saline restricted to the spinal cord. Monosynaptic (monosyn.) events are labeled with an asterisk and polysynaptic (polysyn.) events are labeled with a dot. **(C)** Averaged (mean ± SD) motor response latencies in ipsi- (top) and contralateral (bottom) rostral, medial and caudal Vr evoked by LVST electrical stimulation before and during Ca^2+^/Mg^2+^-enriched saline application. **(D)** Leftmost panel: CNS scheme depicting retrograde (Retro. Dex) labeling of rostral and caudal spinal motoneurons together with anterograde (Antero. dex.) labeling from the LVST. Left picture: confocal stack orthogonal projection of a spinal cross-section showing a retrogradely labeled caudal motoneuron and vestibulospinal terminals anterogradely labeled from the ipsilateral LVST. Middle picture: magnification (×40) of the square area drawn on the left picture with synapsin immunofluorescence labeling. Right picture column: confocal magnification (×60) of the rectangle dashed area drawn on middle picture illustrating, from top to bottom, axial motoneurons dendrites (Ax Mn), vestibulospinal fibers (Vest. Fib.), synapsin (syn.) immunofluorescence, and merge. Fluorescence signal close appositions are pointed with red arrowheads. Scale bars in left and middle pictures are 50 μm and 10 μm, respectively. c.c., central canal; HB, hindbrain; SC, spinal cord.

**Figure 6:**
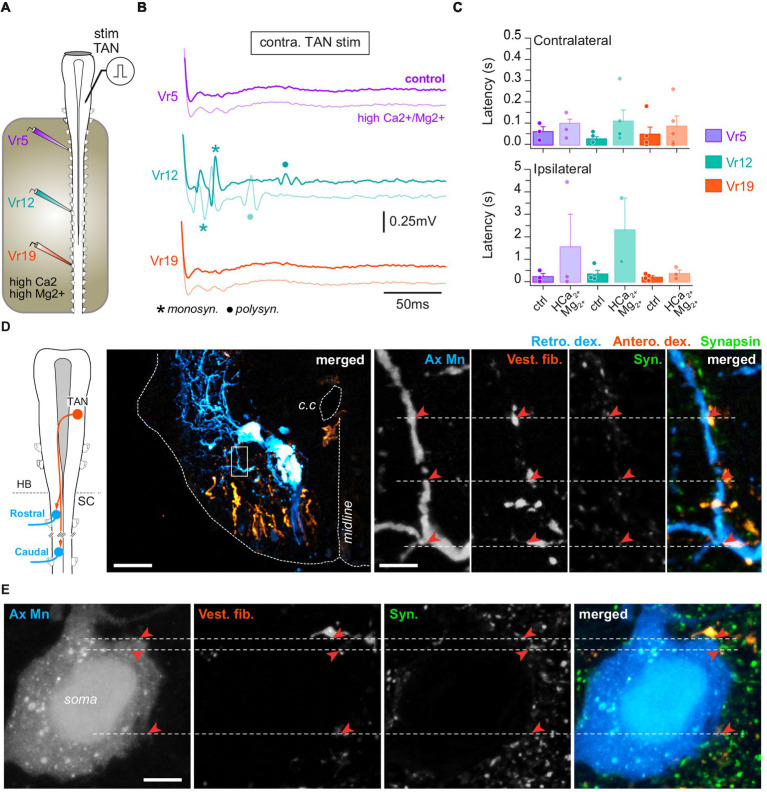
TAN inputs on spinal motoneurons. **(A)** Schematic of the semi-intact preparation used to record spinal ventral root responses to electrical stimulation of the TAN nucleus. **(B)** Trace examples of TAN-evoked motor responses in rostral (Vr5, purple), medial (Vr12, turquoise) and caudal (Vr19, orange) ventral roots before and during bath application of Ca^2+^/Mg^2+^-enriched saline restricted to the spinal cord. Monosynaptic (monosyn.) events are labeled with an asterisk and polysynaptic (polysyn.) events are labeled with a dot. **(C)** Averaged (mean ± SD) motor response latencies in contra- (top) and ipsilateral (bottom) rostral, medial and caudal Vr evoked by TAN electrical stimulation before and during Ca^2+^/Mg^2+^-enriched saline application. **(D)** Leftmost panel: CNS scheme depicting retrograde (Retro. Dex) labeling of rostral and caudal spinal motoneurons together with anterograde (Antero. dex.) labeling from the TAN. Left picture: confocal stack orthogonal projection of a spinal cross-section showing a retrogradely labeled caudal motoneuron and vestibulospinal terminals anterogradely labeled from the contralateral TAN. Right picture series: confocal magnification (×60) of the rectangle area drawn on the left picture illustrating, from left to right, axial motoneurons dendrites (Ax Mn), vestibulospinal fibers (Vest. Fib.), synapsin (syn.) immunofluorescence, and merge. Close signal appositions are pointed with red arrowheads. Scale bars in left picture and magnification are 100 μm and 10 μm, respectively. **(E)** Confocal magnification (×60) of an axial motoneuron soma (Ax Mn), vestibulospinal fibers (Vest. fib.), synapsin (Syn.) immunofluorescence, and merge. Close signal appositions are pointed with red arrowheads. Scale bar is 100 μm. c.c., central canal; HB, hindbrain; SC, spinal cord.

Electrical pulses were used to stimulate directly vestibulospinal cell bodies in the brainstem while motor responses were recorded simultaneously from spinal Vr at rostral, medial and caudal levels (*n* = 10 animals). Focal stimulation of either LVST (Figure 5) or TAN neurons (Figure 6) evoked bursting responses on both sides of the cord, at every spinal level. Stimulation magnitude was then adjusted in order to record minimal responses, typically consisting of short delayed and short duration compound action potentials, sometimes followed a few hundreds of milliseconds later by longer duration bursts.

Single pulse stimulation of LVST neurons (Figure 5A) reliably produced fast compound potentials, with variable delays according to the level of the spinal ventral root (Figure 5B). However, there was a tendency for short latency responses to occur earlier in medial Vr (*n* = 9 animals; Figure 5C; ipsi: 58 ± 30 ms; contra: 88 ± 46 ms) than in rostral (ipsi: 70 ± 35 ms; contra: 110 ± 38 ms) and caudal ones (ipsi: 76 ± 27 ms; contra: 160 ± 62 ms), although differences between grand means were not statistically significant (*p* = 0.66, Kruskal–Wallis test). In addition, LVST-evoked contralateral responses appeared much more variable and occurred with a longer delay than ipsilateral ones (compare upper and lower histograms in Figure 5C), and a similar pattern was generally observed also for late responses (not illustrated). Whereas long latency potentials rapidly faded in saline enriched in divalent cations, fastest responses were preserved, although occurring with a three-fold longer delay (Figure 5C), suggesting the existence of direct synaptic contacts between LVST neurons and spinal MNs.

Unilateral retrograde labeling of spinal MNs was combined with anterograde tracing from the ipsilateral LVST, together with subsequent immunodetection of the presynaptic protein synapsin (Figure 5D). At pre-metamorphic stages, spinal MNs consist of large somata located laterally in the ventral cord, prolonged by long dendritic arborization expanding mainly in the laterodorsal direction (Figure 5D, left image; see also Figure 6D). In the spinal cord, LVST axons were found to run in the ventrolateral white matter [see also (43)], and their terminals to connect spinal MNs at both dendritic and somatic loci (with 73.6% contacts on dendrites; e.g., Figure 5D, middle and right panels). Comparable proportions were obtained in all segments investigated along the spinal cord although the total number of contacts decreased in more distal segments (not illustrated).

Direct electrical stimulation was applied to a TAN nucleus, with the minimal intensity and duration parameters able to evoke reliable responses recorded from spinal ventral roots in normal saline (*n* = 9 animals; Figure 6A). In these conditions, Vr responses were recorded from both sides (Figure 6B) that consisted of short latency compound bursts occurring significantly earlier on the contralateral side in medial (26 ± 10 ms) than rostral (240 ± 180 ms) and caudal segments (48 ± 34 ms; *p* = 0.01, Kruskal–Wallis test; Figure 6C, upper histogram). Response delays were more variable and largely longer on the ipsilateral side in the rostral (230 ± 140 ms) and medial cord (350 ± 160 ms) but not in caudal segments (26 ± 10 ms). As reported above for LVST stimulation, TAN-evoked fast compound bursts persisted under high divalent cation saline (Figures 6B,C), including in ipsilateral ventral roots, although response delays were increased at all levels of the spinal cord (*p* = 0.01, Kruskal–Wallis test).

The persistence of motor responses in saline enriched in divalent cations suggested that some TAN fibers projected directly onto spinal MNs. Retrograde motoneuronal labeling and anterograde tracing of TAN axons were combined to synapsin immunodetection at various levels of the spinal cord (*n* = 8 animals; Figure 6D). TAN axons were found in the same ventrolateral quarter as contralateral LSVT fibers (Figure 6D), and TAN terminals connected spinal MNs principally on dendrites (71.15% of contacts) and, to a lower extent, on soma (Figure 6E). Confocal imaging revealed the presence of close apposition between TAN terminals, MN dendrites and synapsin fluorescent signals in the same focal plane (Figure 6D, right image sequence), which strongly supported the existence of direct synaptic contacts between TAN axons and spinal MNs at all studied rostrocaudal segments.

### Contralaterally-projecting vestibulospinal neurons from a rostral brainstem nucleus activate the spinal swimming network

**Figure 7:**
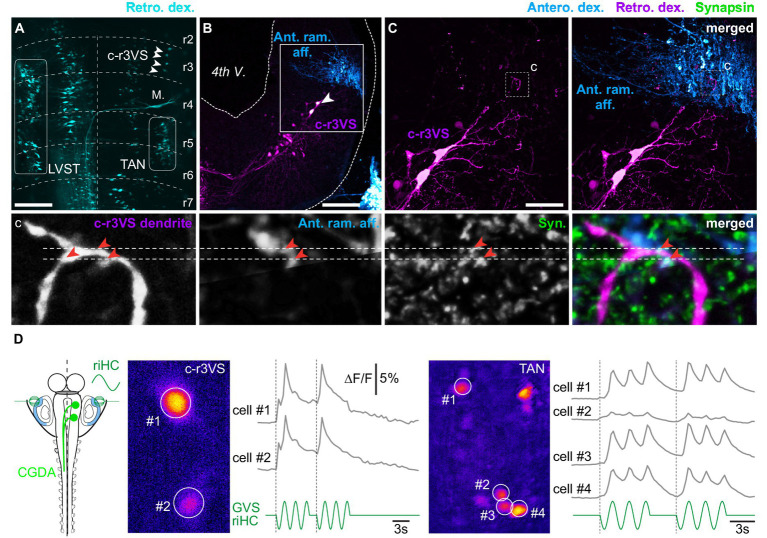
Vestibulospinal neurons located in contralateral rhombomere 3. **(A)** Orthogonal projection of a confocal image stack of a brainstem whole-mount preparation showing vestibulospinal neuron populations retrogradely labeled from the left hemi-cord. White arrowheads indicate vestibulospinal neurons located in the rhombomere 3 (r3), just above the Mauthner cell (M.), and projecting in the contralateral spinal cord (c-r3VS, contralateral rhombomere 3-located vestibulospinal neurons). **(B)** Orthogonal projection of a confocal image stack of a brainstem cross-section showing retrogradely labeled c-r3VS neurons and vestibular terminals anterogradely labeled from the anterior ramus VIIIth nerve branch (Ant. ram. aff.) on the same side. **(C)** Magnifications (×40) of square area in **B**: left panel displays retrogradely labeled c-r3VS neurons only; right panel shows anterogradely labeled anterior ramus vestibular afferents in addition. Bottom picture series display confocal magnifications (×60) of the “c” dashed square area in **C** illustrating, from left to right, dendrites from c-r3VS neurons, vestibular afferent fibers from the anterior ramus nerve branch, synapsin (syn.) immunofluorescence, and merge. Red arrowheads point to close appositions of fluorescent signals. Scale bars in **A–C** pictures are 100 μm, 100 μm and 50 μm, respectively. **(D)** Calcium transients elicited in c-r3VS (left panel, pseudocolor image) and TAN neurons on the same side (right panel) in response to 0.25 Hz GVS sinusoidal current in a semi-intact preparation (left scheme) where vestibulospinal neurons were retrogradely labeled by calcium green dextran-amine (CGDA) and GVS electrode were positioned in order to stimulate horizontal canal cupula. riHC, right horizontal canal cathode.

By the past, a small group of 4–5 brainstem neurons only was described as potentially vestibulospinal neurons, due to their dorsolateral position in rhombomere 3 [(24); Figure 7A]. However, nor their central vestibular identity neither their spinal output was functionally confirmed so far. Unilateral retrograde labeling from a rostral spinal segment confirmed the presence of 3–5 vestibulospinal-like neurons in the contralateral rhombomere 3 (c-r3VS; *n* = 5 animals; Figure 7A). Combined anterograde tracing of VIIIth nerve primary afferents and synapsin immunodetection (Figures 7B,C) showed numerous close appositions of fluorescent signals from sensory afferent, c-r3VS dendrites and synapsin in the same confocal plane (image sequence in expanded inset c), indicating that these spinal-projecting neurons received direct VIIIth nerve sensory inputs. This further suggested that these neurons likely constituted a third nucleus of vestibulospinal neurons that, similarly to TAN neurons, were characterized by axons decussating in the brainstem (Figure 7B) and projecting in the contralateral spinal cord.

Optical recordings of vestibular-evoked calcium transients were performed from TAN and c-r3VS neurons backfilled with calcium green dextran-amine injected in anterior spinal hemi-segments in order to test the functional synaptic connectivity between vestibular afferent terminals and these c-r3VS neurons. Galvanic stimulation of HC cupulas evoked long-duration calcium transients in both c-r3VS and TAN neurons (Figure 7D; respectively, left and right), demonstrating that labyrinthine sensory inputs activated rostral spinal-projecting neurons in a same way as TAN neurons. However, the weaker stimulus-response relationship in c-r3VS neurons (Figure 7D, left) compared to TAN neurons on the same side (Figure 7D, right) suggested that c-r3VS cells received less vestibular inputs. This was also in accordance with VIIIth nerve anterior ramus afferent contacting c-r3VS neuron exclusively on distal dendrites (Figure 7C,c).

Electrical pulses were applied directly in the dorsal region of rhombomere 3 to activate c-r3VS neurons, and ventral root responses were monitored at various levels of the spinal cord in either normal or in divalent cations-enriched saline (*n* = 5 animals; not illustrated). Contrary to TAN or LVST, minimal duration pulses (10 μs) never evoked any reliable response from spinal ventral roots in either saline condition. In contrast, long duration (200 μs) pulses systematically triggered short episodes of fictive locomotion throughout the spinal cord. Swimming episodes did not systematically initiate contralateral to the stimulation side, as one could have expected because of the rostral vestibulospinal fiber decussation, but about 58% of such episodes started on the ipsilateral side. Moreover, the delay of swimming episode onset was affected by the stimulus intensity, whatever the spinal segment considered: the higher intensity, the shorter delay until a plateau was reached for stimulation intensity equal or beyond three times the threshold. In contrast, stimulus intensity did not affect swimming episode parameters such as duration or frequency. Moreover, swimming responses always disappeared in saline enriched in divalent cations, simultaneously at all spinal levels, and no residual burst could be recorded. This latter observation demonstrated the polysynaptic nature of the pathway from c-r3VS neurons to spinal MNs. Altogether, our results suggested this specific descending command to target only the spinal central pattern generator for locomotion.

### Developmental changes of horizontal canal-controlled postural reflex activity

Tadpole stages are temporary developmental steps in an amphibian life, until metamorphosis leads to its adult form for which virtually all physiological systems are reshaped (16). We have previously demonstrated how the vestibulospinal system was organized in the juvenile *Xenopus* and fit the animal’s biomechanics (12). Because the vestibulospinal reflex structure in juveniles was substantially different from that described above in the tadpole, we investigated whether and how tadpole vestibulospinal reflexes evolved from early pre-metamorphosis to pre-climax stages, as a potential blueprint of the adult vestibulospinal organization that becomes functional after metamorphosis climax.

**Figure 8:**
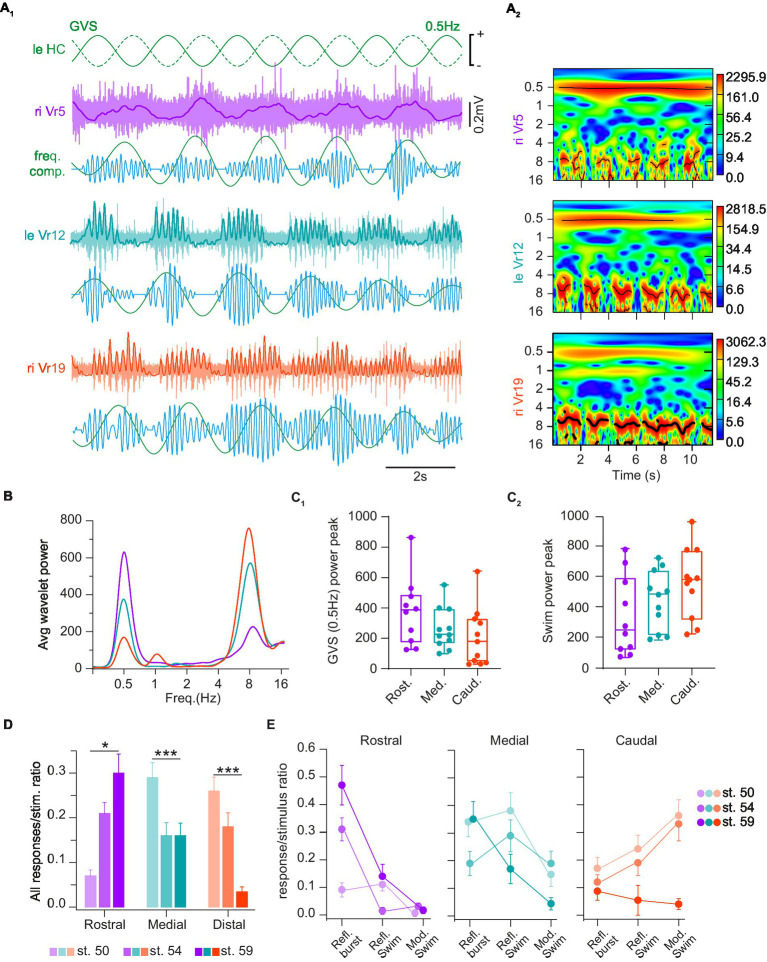
Horizontal canal activation evoked several types of spinal reflex responses. **(A)** Trace examples **(A**_**1**_**)** and corresponding wavelet analysis (wavelet reconstructions in **A**_**1**_ and power spectrum in **A**_**2**_) of vestibulospinal-evoked motor activity in rostral (Vr5, purple), medial (Vr12, turquoise) and caudal (Vr19, orange) right ventral roots (riVr) in response to GVS sinusoidal current stimulating the horizontal canal cupula (HC) at 0.5 Hz. In **A**_**1**_ for each Vr, upper traces show raw and integrated (bold line) nerve recordings, lower traces show wavelet reconstruction at 0.5 Hz (green) and 8 Hz (blue), corresponding to GVS and locomotor frequencies, respectively. **(B)** Periodogram of the average wavelet power for Vr recording example shown in **A**_**1**_. **(C)** Box plots of wavelet power peaks for 0.5 Hz GVS **(C**_**1**_**)** and swimming **(C**_**2**_**)** frequencies, respectively, in rostral (Rost.), medial (Med.) and caudal (Caud.) Vr. Upper and lower error bars represent maximum and minimum values, respectively. Bounds and center lines represent the values of 50% (75 and 25% percentile) and the median values, respectively. **(D)** Ratio of GVS-induced spinal responses of any types in rostral, medial and caudal Vr at larval stage 50, 54 and 59. **(E)** Ratio of GVS-induced reflex bursts (Refl. Burst), reflex swimming short events (Refl. Swim) and vestibular-modulated continuous swimming (Mod. Swim) in rostral (left), medial (middle) and caudal (right) Vr at larval stage 50, 54 and 59. See Table 1 for corresponding statistics.

To this aim, sinewave GVS was bilaterally applied to the HC cupulas, and reflex activity was recorded in rostral, medial and caudal spinal segments, at stage 50 [corresponding to the horizontal canal sensitivity onset (38)], stage 54 [where canal sensitivity is fully mature (35, 38)]; and pre-climax stage 59 [where larval swimming performances diminish and adult-like swimming behaviors rise (28, 35)]. Both the efficiency to evoke reflexes and the nature of these reflexes were evaluated (Figure 8). In larval *Xenopus*, GVS activation of vestibulospinal pathways triggers a continuum of spinal motor reflex responses, from non-rhythmic compensatory postural bursts (as shown in Figures 2, 3) to continuous, vestibular-modulated locomotor sequences. Most of the time GVS-evoked spinal reflex activities were a mixture of motor responses between these two extreme patterns (Figures 8A_1_,A_2_). However, GVS capacity to evoke either postural non rhythmic responses or swimming activity showed tendency to be segmentally different. Vestibular-driven non-rhythmic responses seemed preferentially evoked in rostral spinal segments (Note the continuous red line at 0.5 Hz in the top spectrogram in A_2_; see the large purple peak at 0.5 Hz in Figure 8B and purple box plot in Figure 8C_1_). In contrast, GVS-induced locomotor activities tended to be more frequent in medial and caudal segments (note the thicker red lines around 8 Hz in middle and bottom spectrogram compared to top spectrogram in A_2_; see green and red peaks at 8 Hz in Figure 8B and corresponding whisker boxes in Figure 8C_2_). Although not significant these observations suggested that the vestibular modulation tended to be stronger on rostral spinal circuits and gradually faded in more caudal spinal segments.

GVS-evoked spinal motor responses, either non-rhythmic or locomotor-like, were also differentially expressed over the larva development. Globally, HC-activating GVS became more efficient at eliciting reflex responses in rostral spinal segments in pre-metamorphosis and metamorphosing tadpoles (*p* < 0.001; Figure 8D, purple bars). Conversely, distal spinal responses were harder to evoke in older larvae (*p* < 0.001; Figure 8D, orange bars). Finally, medial responses tended to remain stable over tadpole development although the ratio of evoked response per stimulus cycle was slightly higher in younger animals (*p* < 0.05; Figure 8D, green bars). More specifically, in rostral segments (Figure 8E, purple graph) non-rhythmic postural reflexes were much less frequent in the youngest larvae compared to just before or during metamorphosis (*p* < 0.001), whereas no statistical differences were found considering the two swimming-related reflex responses. In caudal segments (Figure 8E, orange graph) GVS-induced spinal responses in young larvae preferentially corresponded to swimming activity, a characteristic that was lost in the metamorphosing tadpole (*p* < 0.001). In contrast, no significant stage-dependent changes in reflex response were observed in spinal medial segments (Figure 8E, green graph); however, reflex bursts occurred significantly more often in both the younger (*p* = 0.035) and the older tadpoles (*p* = 0.012). All statistical results about response type and developmental stages are presented in Table 1.

**Table 1:** Developmental changes in vestibulospinal reflex expression - Statistics.

Rostral		Stage 50			Stage 54			Stage 59		
		Refl.	Refl.	Mod.	Refl.	Refl.	Mod.	Refl.	Refl.	Mod.
		Burst	Swim	Swim	Burst	Swim	Swim	Burst	Swim	Swim
St. 50	RB		ns	ns	***			***		
	RS	0.999		ns		ns			ns	
	MS	0.482	0.212				ns			ns
St. 54	RB	<0.001				***	***	*		
	RS		0.312		<0.001		ns		ns	
	MS			0.999	<0.001	0.999				ns
St. 59	RB	<0.001			0.013				***	***
	RS		0.999			0.123		<0.001		ns
	MS			0.999			0.999	<0.001	0.224	

Medial		Stage 50			Stage 54			Stage 59		
		Refl.	Refl.	Mod.	Refl.	Refl.	Mod.	Refl.	Refl.	Mod.
		Burst	Swim	Swim	Burst	Swim	Swim	Burst	Swim	Swim
St. 50	RB		ns	ns	ns			ns		
	RS	0.999		*		ns			ns	
	MS	0.035					ns			ns
St. 54	RB	0.451				ns	ns	ns		
	RS		0.934		0.885		ns		ns	
	MS			0.999	0.999	0.885				ns
St. 59	RB	0.999			0.504				ns	*
	RS		0.156			0.831		0.457		ns
	MS			0.908			0.627	0.012	0.857	

Distal		Stage 50			Stage 54			Stage 59		
		Refl.	Refl.	Mod.	Refl.	Refl.	Mod.	Refl.	Refl.	Mod.
		Burst	Swim	Swim	Burst	Swim	Swim	Burst	Swim	Swim
St. 50	RB		ns	ns	ns			ns		
	RS	0.972		ns		ns			ns	
	MS	0.077	0.619				ns			***
St. 54	RB	0.997				ns	*	ns		
	RS		0.997		0.972		ns		ns	
	MS			0.999	0.032	0.406				**
St. 59	RB	0.959			0.999				ns	ns
	RS		0.177			0.589		0.999		ns
	MS			<0.001			0.002	0.999	0.999	

*p < 0.05; **p < 0.01; ***p < 0.001.

## Discussion

In this study, we investigated the functional organization of vestibulospinal pathways involved in postural activity in the pre-metamorphic tadpole. We showed that head rotations in the horizontal plane are the most effective to trigger reliable spinal reflex responses, a stimulus-response relationship that is well mimicked by HC galvanic stimulation. GVS-evoked spinal motor responses were present at every level of the cord, but did not depend on the initial activation of rostral segments (which will be preserved after metamorphosis). We further demonstrated that such reflex responses relied on various populations of vestibulospinal neurons projecting either ipsi- or contralaterally in all spinal segments and, at least for a part, directly onto spinal MNs. Finally, we showed that GVS-evoked spinal responses gradually evolved during larval development, likely towards postural reflexes comparable to those previously described in post-metamorphosis animals (12).

The type of vestibular endorgan activated strongly shaped the response pattern of the postural spinal activity in larvae. Whereas horizontal head movements (or GVS selective HC activation) were reliably able to trigger phase-linked, organized motor bursts along the spinal cord, head movements imposed in the two vertical planes (pitch and roll) mostly evoked more or less phase-modulated tonic firings in ventral roots (Figures 1, 2). On the one hand, such an apparently simple postural modulation in response to vertical head movements may result from the fact that both the branches innervating dorsal and ventral tail muscles were recorded simultaneously in our experiments, which certainly not allowed us to discriminate a more complex pattern of response, as previously reported in the larval zebrafish (44). On the other hand, qualitative differences between horizontal and vertical stimulation-evoked reflex activity could also be functionally related to the tadpole’s postural and swimming behaviors. Swimming behavior is based on tail undulations that engender cyclic lateral displacements of the head, thus systematically activating horizontal semicircular canals predominantly. In turn, canal activation produces the necessary phase-linked postural adaptation, as well as possible swimming enhancement (45, 46). Meanwhile, vertical semi-circular canals and otoliths are not or much less involved during swimming, and their role may be restricted to provide intermittent postural adjustment signals during fast buoyancy or direction changes in the water column. In the absence of active locomotion, larvae maintain a stable, almost vertical, pitch down position (head oriented downward) in the water column, and are mostly submitted to rolling due to water dynamics. In these conditions the two vertical semi-circular canals and, above all, the otoliths play a crucial role in detecting small changes of the body orientation. Then, the resulting vestibulospinal command should generate slight variations of muscle tonicity to compensate for small amplitude postural deviations. In contrast, activation of horizontal canals should result from large amplitude disequilibrium, which even could likely require a swimming-like activity to restore body balance (or flee away from the source of the disequilibrium) evoked by strong phasic vestibulospinal commands.

Although the anatomical arrangement of the central vestibular nuclei directly implicated in posture control is globally well conserved in vertebrates, few differences among species were identified in the past. Hence, vestibulospinal postural commands emanate from two main groups of neurons, one located very lateral in the fourth ventricle and the other being located more medially, closer to the reticulospinal nuclei (47). Whereas in mammals the medial nucleus gives rise to projections traveling bilaterally in the spinal cord, the TAN nucleus in terrestrial frogs (location equivalent of the mammalian medial nucleus) only sends contralateral projections into the spinal cord (24, 48). In *Xenopus*, the two same main nuclei as in terrestrial frogs were described in both juvenile and larvae (12, 23). However, we additionally demonstrated here that the lateral nucleus (LVST) comprised two distinct vestibulospinal neuron populations characterized by distinct projection pathways (Figure 4). Especially, a population located in the medial part of the LVST nucleus sends projections in the medial vestibulospinal tract, which was considered to convey only TAN fibers (and reticulospinal axons) in frogs (23, 24, 48). Although this possibility has been briefly considered in a recent review of the literature (47) we provide the first demonstration of such an organization in the larval frog. Thus, our findings suggest a common organization of vestibulospinal pathways to exist in amphibians, mammals and birds, where such medially-located LVST neurons in larval *Xenopus* may be comparable to the mammalian and avian ipsilateral medial vestibulospinal tract neurons, but without a clear segregation from the classical laterally-projecting LVST neurons.

We identified another population of contralaterally-projecting vestibulospinal neurons (c-r3VS neurons) that were located very rostral in rhombomere 3, at the same level as the most rostral LVST neurons (Figure 7). These neurons were previously proposed as putative vestibular neurons in amphibians but without a real demonstration of their activation by vestibular sensory inputs. Contrary to LVST and TAN cells the stimulation of these neurons, which we have shown to receive excitation from vestibular sensory afferents, was not able to produce simple spinal reflexes but consistently triggered episodes of swimming activity. Such a response pattern may question the nature of c-r3VS neurons, since no links could be found between the intensity of sensory stimulation and the graduation of the motor response, which characterize adaptation-related sensory-motor systems (49). Rather, c-r3VS neurons acted as escape command neurons. Reticulospinal neurons, likely homologs to Mauthner cells, have been previously described in the zebrafish (50) that responded to vestibular inputs to trigger escape swimming behaviors; however, their location in brainstem was totally different from the c-r3VS neuron site described in the present study. In contrast, a population of pontine reticulospinal neurons were also reported in the mouse (51, 52), with a location similar to c-r3VS neurons in the *Xenopus* tadpole, whose stimulation triggers rapid spinal MN responses. However, despite their location near the most rostral vestibular sensory afferent terminals, it is not known whether pontine reticulospinal cells process vestibular inputs as we have shown here for c-r3VS neurons.

As in the juvenile (12), we report here that larval vestibulospinal neurons from the two main nuclei (LVST and TAN) both send projections into the caudal spinal cord (Figure 3). More surprisingly, we found that the large majority of vestibulospinal neurons projected into the spinal segments that were destined to degenerate during metamorphosis, whereas only one third of the labeled neurons stopped within the first 10 spinal segments [the segments that will be preserved during metamorphosis and ensure all postural and locomotor functions (12, 27)]. Although we cannot exclude the possibility that persisting collaterals from these far-projection neurons may play a role in post-metamorphic animals, this observation suggests a strong reorganization of the vestibular inputs on spinal motor networks to occur during metamorphosis.

Power analysis (Figures 8A–C) clearly identified two main frequency components in GVS-induced spinal motor responses: one component at the stimulation frequency (burst responses at 0.5 Hz in the figure example), and one component at the fictive swimming frequency (around 8 Hz). However, closer analysis of activity patterns (Figures 8D,E) identified two swimming-related reflex responses; one was characterized by discontinuous swimming bouts occurring at GVS peaks and interrupted in between, whereas the other was a continuous fictive swimming showing GVS-related cyclic modulation in swim burst amplitude. The occurrence of the three distinct forms of vestibulospinal reflex response appeared to depend both on the rostro-caudal spinal segment position and the larval stage. Whereas rostral spinal segments produced GVS phase-related cyclic bursts more reliably than swimming-like responses at all developmental stages, more caudal segments systematically generated swimming-like responses in pre-metamorphic animals (stages 50 and 54). Moreover, the more distal the segments were, the more often continuous swimming occurred with GVS-related cyclic modulation. Metamorphosing animals (stage 59) were characterized by a distinct response pattern where caudal spinal segments showed very few reflex responses, and the rostral/medial segments preferentially expressed GVS phase-related bursts. Thus, during tadpole maturation rostral segments gradually acquired a stronger ability to generate GVS-related postural bursts while distal segments progressively lost their capacity to respond, prefiguring so, the pattern of GVS-evoked postural reflexes reported in post-metamorphic frogs (12).

## Data availability statement

The raw data supporting the conclusions of this article will be made available by the authors, without undue reservation.

## Ethics statement

The animal study was approved by CEEA50 Comité d’éthique pour l’expérimentation animale Bordeaux. The study was conducted in accordance with the local legislation and institutional requirements.

## Author contributions

GB: Formal analysis, Investigation, Writing – original draft. AO-B: Formal analysis, Investigation, Writing – review & editing. MP: Investigation, Writing – review & editing. JB-C: Formal analysis, Methodology, Writing – review & editing. LC: Investigation, Methodology, Writing – original draft. M-JC: Investigation, Methodology, Writing – original draft. DLR: Conceptualization, Formal analysis, Funding acquisition, Investigation, Methodology, Supervision, Writing – original draft, Writing – review & editing. FL: Conceptualization, Funding acquisition, Investigation, Methodology, Project administration, Supervision, Writing – original draft, Writing – review & editing.

## Footnotes

^1^ https://fiji.sc/
